# Supplementary material for: Spin–orbit torque switching in a T-type magnetic configuration with current orthogonal to easy axes
Source: Nat Commun. 2019 Jan 16;10:233. doi: 10.1038/s41467-018-08181-y (PMC6335407; doi:10.1038/s41467-018-08181-y)
Supplement: Supplementary file 1 — Supplementary Information [file 41467_2018_8181_MOESM1_ESM.pdf]

Supplementary information

Spin-orbit torque switching in a T-type magnetic configuration with current orthogonal to easy axes

Kong et al.

Outlines

Supplementary Note 1: Irrelevance of Type-T switching with sequences how current is applied (Supplementary Figure 1)

Supplementary Note 2: Model of Type-z switching mode (Supplementary Figure 2-6)

Supplementary Note 3: Model of Type-T switching mode (Supplementary Figure 7-12)

Supplementary Note 4: Immunity of Type-T mode to external fields (Supplementary Figure 13-14 and Supplementary Table 1)

Supplementary Note 1: Irrelevance of Type-T switching with sequences how current is applied

We have implemented the experiment to rule out the possibility of toggle switching mechanism. At zero bias field, current induced magnetization switching is examined by opposite current switching orders +*I* → -*I* → +*I* (+-+ for short) and -*I* → +*I* → -*I* (-+- for short). The results are shown in Supplementary Fig. 1. Independent on current scanning orders, the switching polarity remains unchanged, indicating the switching is deterministic other than toggle switch process. This switching experiment is reproduced for six times. The critical current shows little random fluctuations around 36 mA.

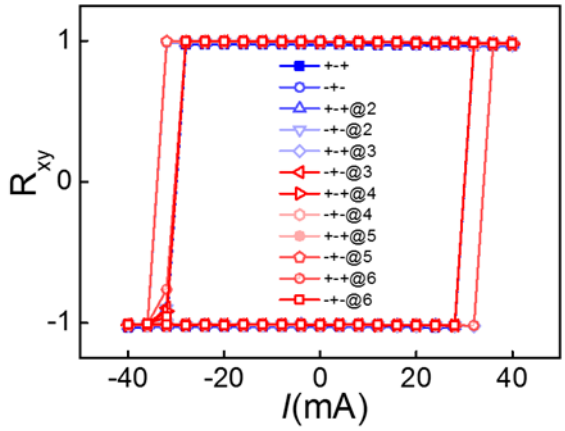

Supplementary Figure 1. Six circles of magnetization switching with opposite current scanning orders (<+-+> and <-+->). The switching polarity remains unchanged under current in different orders.

Supplementary Note 2: Model of Type-z switching mode

We first analyze Type-z mode: Easy axis (EA) of the IMA layer and current I_x are both along the x axis. Experiments show two features: (1) opposite magnetizing history of the IMA layer reverses switching direction of the PMA layer and (2) there is remarkably negative offset in R_{xy} vs. I_x hysteresis loops. The following model with an assumption of tilt EA of the PMA layer toward the x axis (EA of IMA layer) can account for both features. System energy E_T is composed by effective anisotropies of both layers, antiferromagnetic coupling and Zeeman splitting. (Supplementary Equation 1)

$$E_{\mathrm{T}} = E_{\mathrm{OP}} + E_{\mathrm{IP}} + E_{\mathrm{IEC}} + E_{\mathrm{Z}}$$

$$E_{\mathrm{OP}} = -K_1 \cos^2 \beta$$

$$E_{\mathrm{IP}} = -K_2 \sin^2 \theta_2 \left[\eta + (1-\eta) \sin^2 \varphi_2\right]$$

$$E_{\mathrm{IEC}} = A[\sin\theta_1 \sin\theta_2 \cos(\varphi_1 - \varphi_2) + \cos\theta_1 \cos\theta_2]$$

$$E_{\mathrm{Z}} = -HM_1 \sin\theta_1 \cos(\varphi_1 - \varphi_{\mathrm{H}}) - HM_2 \sin\theta_2 \cos(\varphi_2 - \varphi_{\mathrm{H}})$$

Here θ and ϕ are polar and azimuth angle in spherical coordinates, respectively. EA and magnetization of the PMA layer is ($\sin\theta_0\cos\phi_0$, $\sin\theta_0\sin\phi_0$, $\cos\theta_0$) and ($\sin\theta_1\cos\phi_1$, $\sin\theta_1\sin\phi_1$, $\cos\theta_1$), respectively. Angle β between the EA and the PMA layer is thus defined by $\cos\beta = \sin\theta_1 \sin\theta_0 \cos(\varphi_1 - \varphi_0) + \cos\theta_1 \cos\theta_0$. Magnetization of the IMA layer is ($\sin\theta_2\cos\phi_2$, $\sin\theta_2\sin\phi_2$, $\cos\theta_2$). K_1 and K_2 are effective PMA and IMA energies, respectively. Parameter 1-η denotes the ratio of in-plane uniaxial anisotropy to total in-plane anisotropy. A is antiferromagnetic coupling constant. H is applied field. M_1 and M_2 are saturated magnetization of the PMA and IMA layers, respectively. ϕ_{H} is azimuth angle of external field. ϕ_{H}=0° for +x axis and ϕ_{H}=90° for +y axis.

I_x in Ta induces spin current with polarization along the y axis via spin Hall Effect. Then spin currents are transferred up and down to both ferromagnetic layers and activate their spin dynamics and switching. The two ferromagnetic layers sandwich the Ta layer. Thus spin currents absorbed by them have opposite signs. Spin current σ has dimension of K_1/M_1 in this model. As provided spin currents are not too high, equilibrium magnetizations of both layers are constrained in XOZ plane. In this case, the final state of the system can be obtained via torque equilibrium condition Equation. (Supplementary Equation 2)

$$K_1 \sin 2(\theta_1 - \theta_0) - A\sin(\theta_1 - \theta_2) - HM_1 \cos\theta_1 - a_1\sigma = 0$$

$$K_2 \sin 2\theta_2 - A\sin(\theta_1 - \theta_2) + HM_2 \cos\theta_2 - a_2\sigma = 0$$

The parameter $a_{1/2}$ characterizes interfacial absorption efficiency of spin current for the PMA/IMA layer. Further from the above Equation, it is straightforward to obtain an Eigen Equation which determines system stability and evolution routes. (Supplementary Equation 3)

$$\begin{bmatrix} \mathrm{d}\theta_1 \\ \mathrm{d}\theta_2 \end{bmatrix} = \Pi^{-1} \begin{bmatrix} a_1 \\ a_2 \end{bmatrix} \mathrm{d}\sigma$$

$$|\Pi| = [2K_1 \cos 2(\theta_1 - \theta_0) + HM_1 \sin\theta_1 - A\cos(\theta_1 - \theta_2)][2K_2 \cos 2\theta_2 - HM_2 \sin\theta_2 + A\cos(\theta_1 - \theta_2)] + A^2 \cos^2(\theta_1 - \theta_2)$$

$$\Pi^{-1} = \frac{1}{|\Pi|}\begin{bmatrix} [2K_2\cos 2\theta_2 + A\cos(\theta_1-\theta_2) - HM_2\sin\theta_2] & -A\cos(\theta_1-\theta_2) \\ A\cos(\theta_1-\theta_2) & [2K_1\cos 2(\theta_1-\theta_0) - A\cos(\theta_1-\theta_2) + HM_1\sin\theta_1] \end{bmatrix}$$

System is stable as $|\Pi|<0$ and becomes unstable as $|\Pi|$ approaching 0. We are especially interested in the case of zero external field. In order to derive switching behaviors, we have to first obtain initial states at $\sigma=0$ and then apply deduced Equation 3 to move system toward final states at σ with iterative algorithm.

There are 4 initial states available at zero current. They are named as A, B, C and D as shown below. We have used the following set of parameters here as well as in the coming calculations. $K_1=1$, $K_2=2$, $A=0.6$, $\eta=0.5$, $M_1=1$; $M_2=0.6$, $\theta_0=-10^o$, $a_1=1$; $a_2=0.6$.

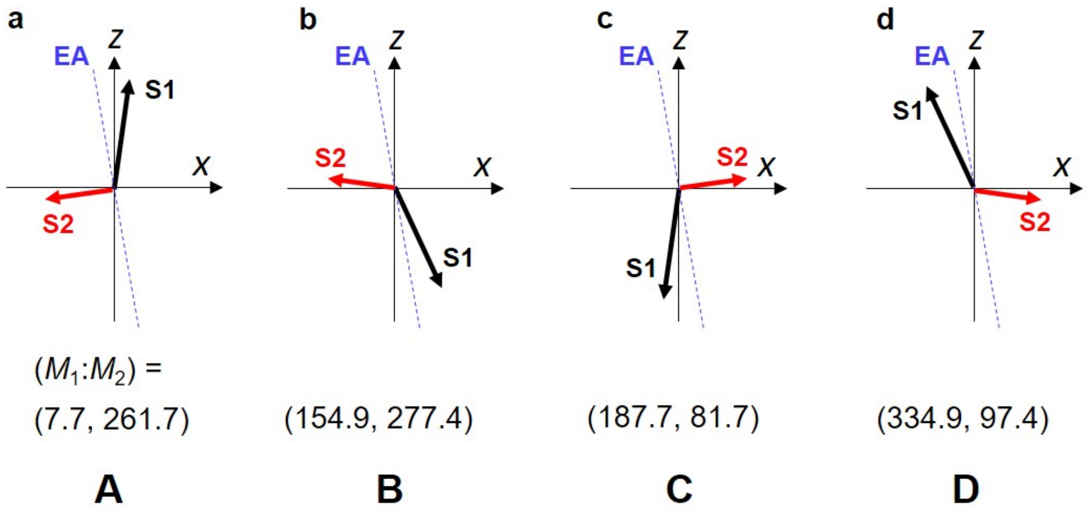

Supplementary Figure 2. Available initial states at zero current with the above set of parameters. The red and black arrows donate the spins of IMA and PMA layer accordingly. The blue dashed line indicates the titling EA of PMA layer.

Torque (denoted by σ) dependence of determinant of the Eigen matrix $|\Pi|$ is shown in Supplementary Fig. 3. Two features are worth highlighting. First, States A and C (States B and D) share the same torque region where both of them are stable. Out of the region they become unstable simultaneously. It means transitions A↔C and B↔D are forbidden. Second, critical torques to induce transitions at positive and negative directions are distinct ($\sigma_1 \neq \sigma_2$), indicating offset M vs σ curves.

In order to further look into switching route, we have calculated critical torque σ_c as well as spin configurations (θ_{1c}, θ_{2c}) at the corresponding transition point which is integrated from torque equilibrium condition Equation with H=0. Parameters $\varepsilon_{1/2}$ with energy dimension are mathematically constructed to help stability analysis at transition points. Minimum locations in ε_i vs. θ_i curves indicate steady state at a transition point. The ε_i vs. θ_i curves in Supplementary Fig. 4 can thus tell switching route. (Supplementary Equation 4)

$$\varepsilon_1 = -\frac{1}{2}K_1 \cos 2\,(\theta_1 - \theta_0) + A\cos(\theta_1 - \theta_{2c}) - a_1\sigma_c\theta_1$$

$$\varepsilon_2 = \frac{1}{2}K_2 \cos 2\,\theta_2 + A\cos(\theta_{1c} - \theta_2) + a_2\sigma_c\theta_2$$

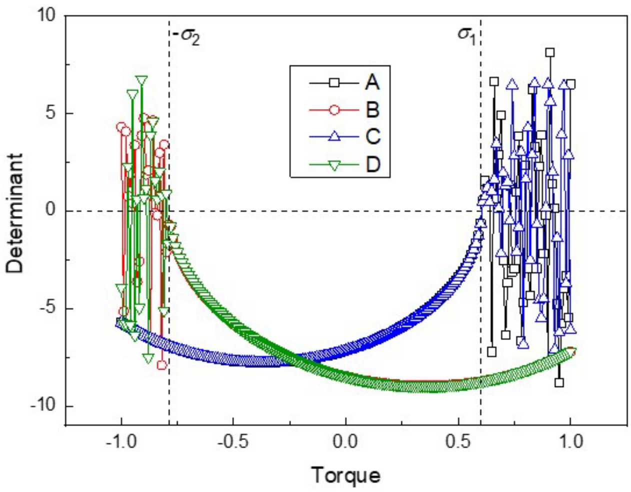

Supplementary Figure 3. Torque dependence of determinant |*Π*| with different initial states A, B, C and D, respectively. The noise data at large torques (outside the dashed lines at $-\sigma_2$ and $+\sigma_1$) after the determinant goes cross zero suggest states under this condition are unstable. Only those states with their determinant smaller than 0 are stable, which should continuously change with torque.

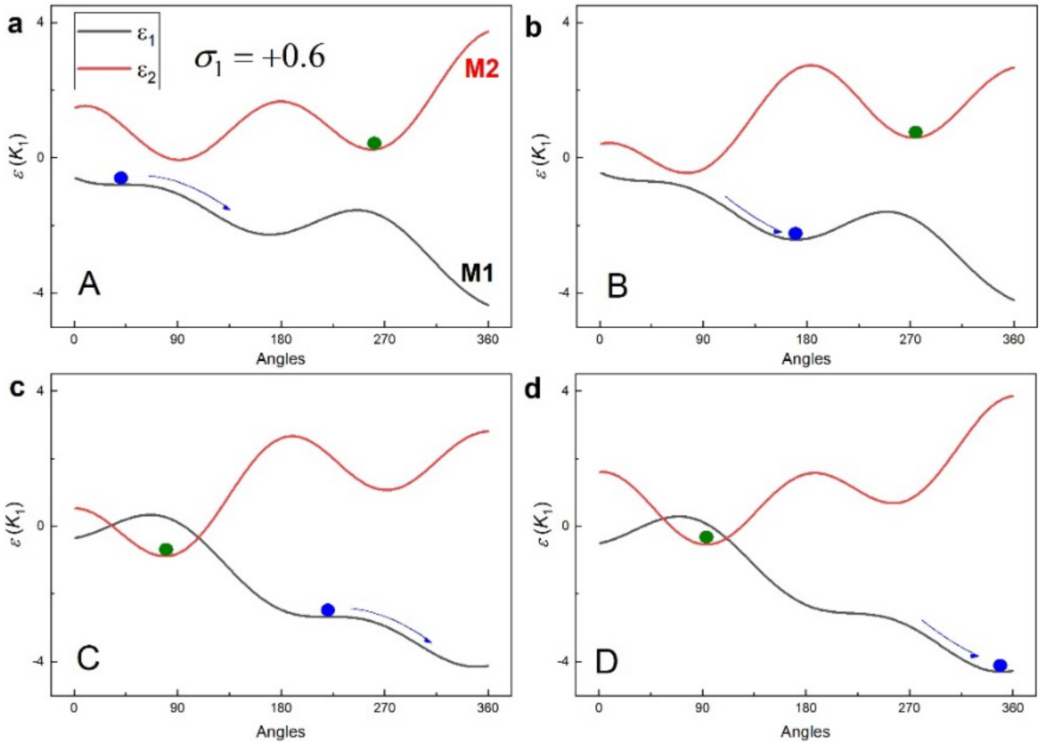

Supplementary Figure 4. Stability analysis of State A, B, C and D at σ=0.6. The green and blue balls in (**a-d**) indicate the stable states of θ_2 or transition routes of θ_1 accordingly. Initial and final states of θ_1 are also highlighted by the blue arrows.

For example, State A turns unstable at σ=0.6. We can see from Supplementary Fig. 4a that instability of system is only brought about by the PMA layer (**M**1) whose stable positions are indicated by blue dots. Supplementary Fig. 4c-d shows very different stable positions (green dots) of IMA layer (**M**2) with Supplementary Fig. 4a-b, indicating neither A↔C nor A↔D transitions in this case are feasible because the IMA layer is in stable position and cannot switch. In fact, Supplementary Fig. 4a-b shows M_1 can naturally slip from State A into State B at σ_1=0.6. According to the above analysis, we can conclude transition A↔B is the only permitted switching route for the system as State A becomes unstable. Similar case occurs for the transition C↔D.

Therefore the final switching diagram in Type-z mode can be depicted by Supplementary Fig. 5. There are three noticeable characteristics in the diagram. (1) M_{1z} switches while M_{2x} retains its orientation during transitions. (2) Switching direction of M_{1z}, clockwise or counterclockwise, depends on direction of M_{2x}, along the -x or +x

axis, respectively. (3) There are apparently the same offsets of M_{1z} vs. torque (denoted by σ) hysteresis loops in both Transitions A↔B and C↔D. Offset direction depends on tilting angle of EA of the PMA layer. For example, negative offset results from negative tilt angle. Characteristics (1) and (2) have been reported in similar structures by Wang et al[1] and Baek et al[2]. The 3rd characteristic is unique here, which is induced by EA tilting of the PMA layer toward proper direction (toward the x axis here). No offset is predicted if the tilt is absent.

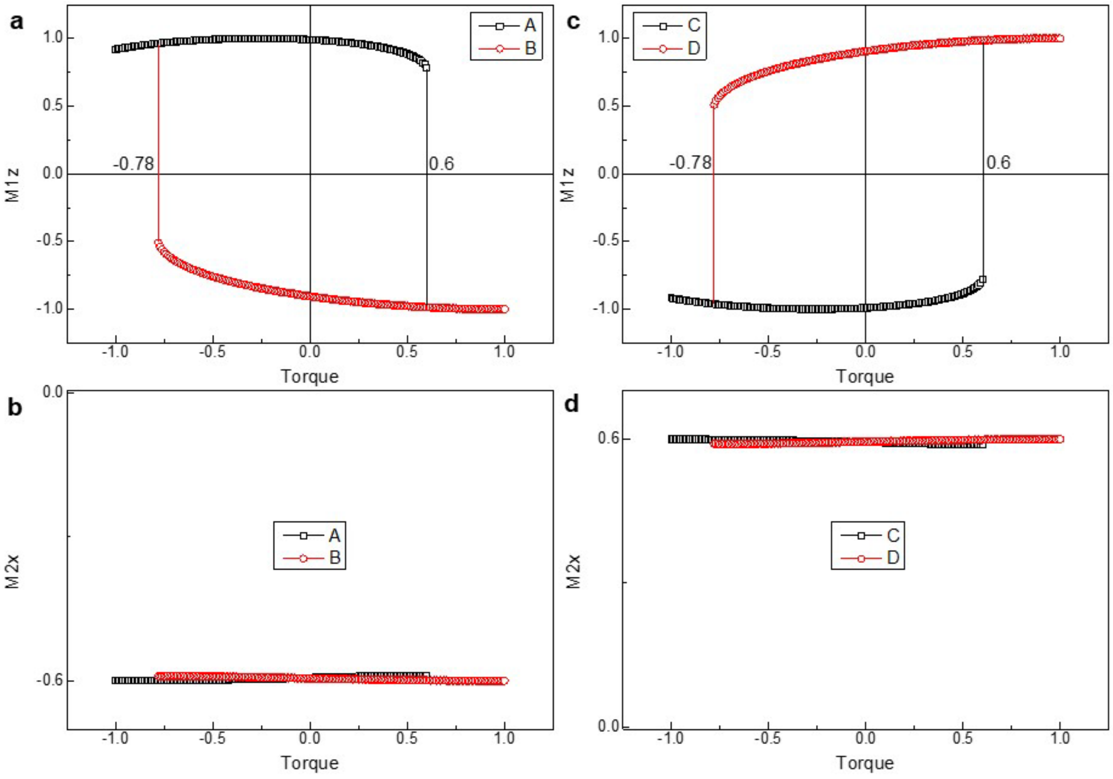

Supplementary Figure 5. *M* vs. torque switching loops in Type-z mode of PMA-IMA coupled system with EA of the PMA layer tilting counterclockwise toward the x axis by a small angle. The parameters in Supplementary Fig. 2 are adopted here. Torque dependence of (**a**, **c**) M_{1z} and (**b**, **d**) M_{2x} indicate the transition A↔B (**a**, **b**) and C↔D (**c**, **d**).

Finally, by evaluating offset in *M*-*I* curves of Type-z mode, we can obtain the tilting angle in our case to be about 10° as shown in Supplementary Fig. 6. As sketched by Supplementary Fig. 6a, it is clear that the offset becomes gradually larger as the tilting angle of the PMA layer becomes larger. We can read out J_{c+} and J_{c-}. J_c is proportional

to σ_c. Then we can obtain $J_c=(J_{c+}-J_{c-})/2$, $J_{ex}=(J_{c+}+J_{c-})/2$ and J_{ex}/J_c as shown in Supplementary Fig. 6b. The ratio of J_{ex}/J_c monotonically increases with the tilting angle and can be used as marker to evidence the tilting angle of a real T-type system. The dotted line in Supplementary Fig. 6b indicated the experimental value retrieved from Figure 2d in the main text, which means the tilting angle of the PMA layer in our T-type structure is around 10°.

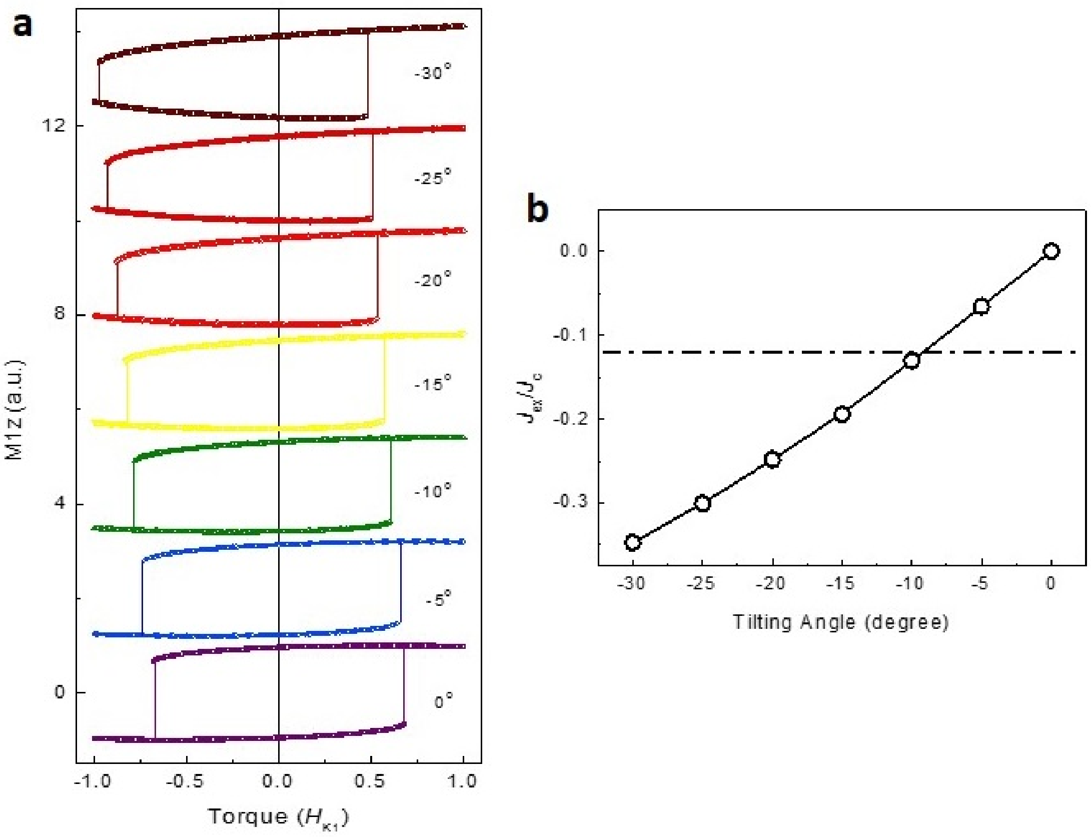

Supplementary Figure 6. The determination of tilting angle. (**a**) Simulated dependence of M_{1z} on SOT in Type-z mode under different tilting angles. (**b**) Derived J_{ex}/J_c as a function of the tilting angle. The dashed line shows the experimental value.

Supplementary Note 3: Model of Type-T switching mode

Significance of this paper is contributing to spin-orbitronics a new switching scheme of PMA materials with robust external-field immunity. Distinguished from classic Type-z mode discussed above [1, 2], in-plane current is applied perpendicularly to EA of the IMA layer in this new scheme. In the following, we will prove the scheme is physically feasible and the deterministic switching can be reproduced if EA of the PMA

layer tilts properly. Coordinate system in Type-T mode is different from that in Type-z mode: current is applied along the x axis while EA of the IMA layer is aligned along the y axis. In this case, EA of the PMA layer should also tilt toward the y axis (also EA of IMA layer here). The same parameters in Supplementary Fig. 2 are adopted. We can also obtain 4 states at I_x=0 in Supplementary Fig. 7, which are virtually the same with those in Supplementary Fig. 2.

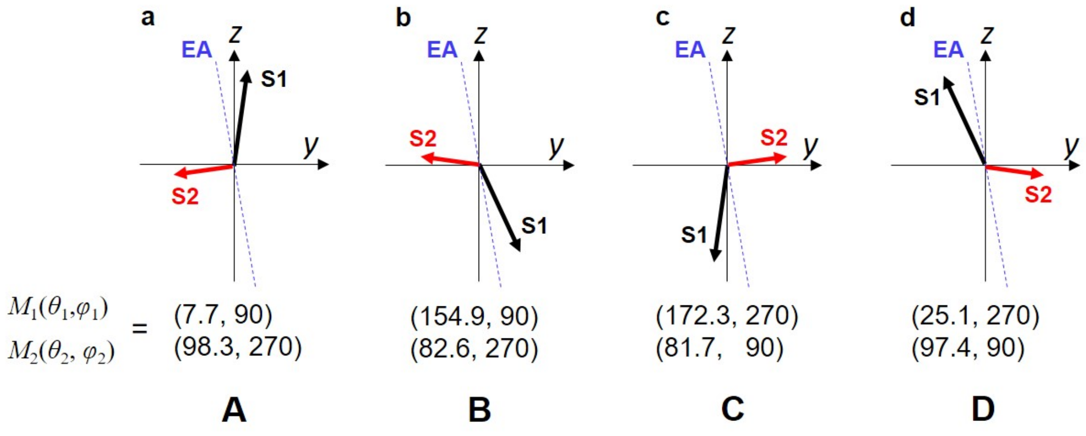

Supplementary Figure 7. Initial states available in Type-T mode as I_x=0 and H=0. They are essentially the same with those in Supplementary Fig. 2. Only difference is that the x axis in Supplementary Fig. 2 is replaced by the y axis in Supplementary Fig. 7 due to reset of measurement setups in Type-T mode.

To analyze Type-T mode, we have to adopt time-dependent LLGS Equation. (Supplementary Equation 5)

$$\frac{\partial \vec{m}_i}{\partial t} = -\gamma \vec{m}_i \times \vec{H}_{i,\mathrm{eff}} + (-1)^{i+1} a_i \vec{m}_i \times \vec{\sigma} \times \vec{m}_i + \alpha \vec{m}_i \times \frac{\partial \vec{m}_i}{\partial t}$$

Here $\mathbf{H}_{i,\mathrm{eff}}$ is effective field experienced by the ith layer, 1st and 2nd layer for PMA and IMA layer, respectively. $\mathbf{H}_{i,\mathrm{eff}}$ can be easily obtained from energy Equation. The parameter α is damping constant and set as 0.1. The parameter a_i characterizes interfacial absorption efficiency of spin current for the ith layer. After I_x is injected or polarization of spin current σ with dimension of K_1/M_1 is produced along the y axis, spin dynamics of the system is activated. The final steady states started from different initial states under variable torques (denoted by σ) are summarized in Supplementary

Fig. 8.

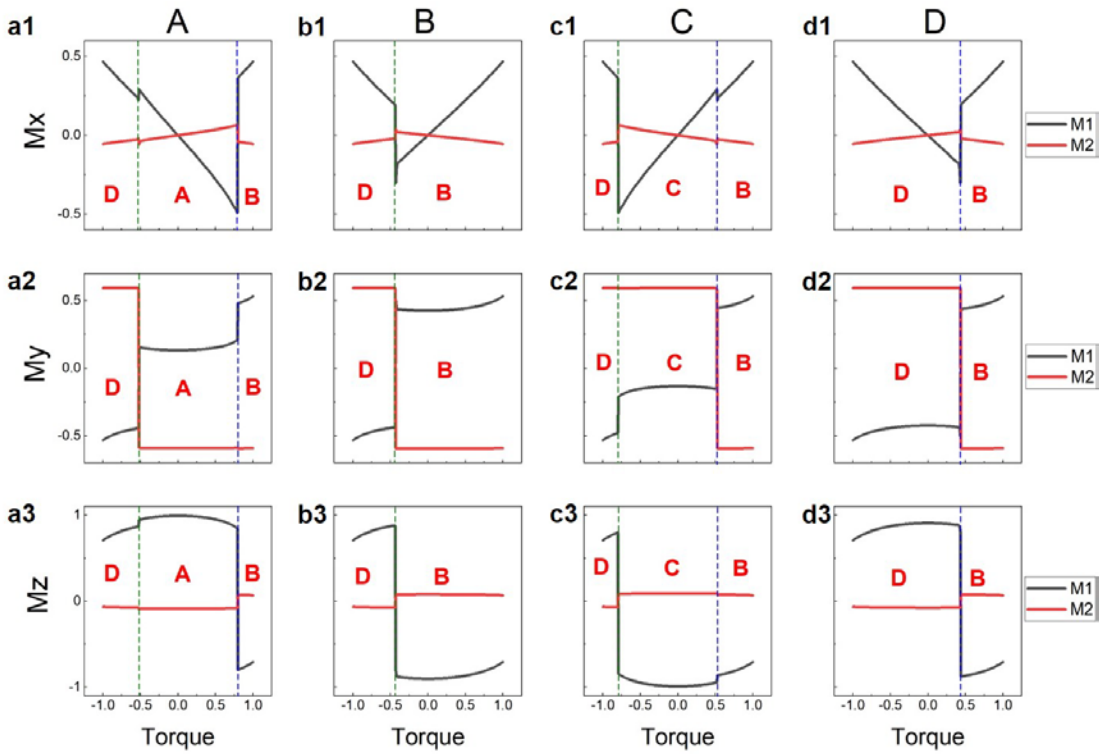

Supplementary Figure 8. Final steady states (denoted by red characters as insets) started from the 4 initial states (marked by black characters on top) driven by torque. (**a**$_i$-**d**$_i$) for initial State A-D. Dashed lines indicate transition from one state to another. After applying large enough torque, only transition B↔D becomes visible or in other words States B and D are dynamically reachable even though system initially locates at State A or C.

Supplementary Fig. 8 tells that system will be finally stabilized into State D and B, respectively, at large enough negative and positive torques, even though it initially locates at State A or C. State A and C turn unstable at large enough positive and negative torques. Therefore, the transition B↔D will become the only permitted switching route driven by torque in this case as shown by Supplementary Fig. 9. The following points with comparison with Type-z mode are worthy of special attentions. (1) Type-T mode switches both IMA and PMA layers simultaneously while Type-z mode switches PMA layer only. (2) Switching direction (clockwise) in Supplementary Fig. 9a is determined by EA tilting angle of the PMA layer. Here we have used θ_0=-10° (as shown in

Supplementary Fig. 7). If we use θ_0=+10°, switching direction of the M_{1z} vs. torque loop is also changed to counterclockwise (Supplementary Fig. 10a). In this sense, there should be a fixed relation between switching direction in Supplementary Fig. 9a (or Figure 3c in main text) and offset direction in Supplementary Fig. 5a,c (or Figure 2c,d in main text).

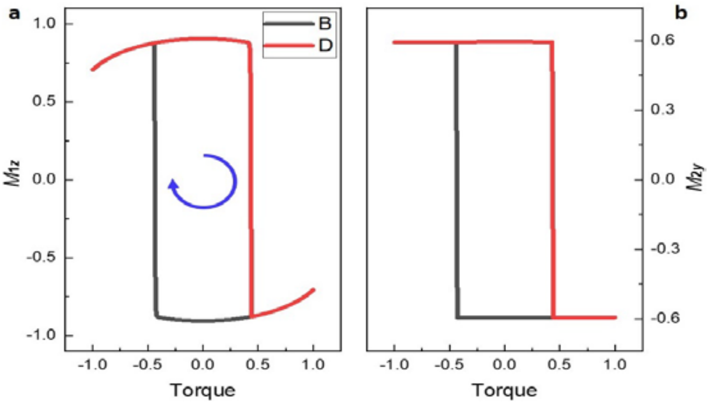

Supplementary Figure 9. The switching chirality when the titling angle is -10°. See switching curves of (**a**) M_{1z} and (**b**) M_{2y} driven by spin-orbit torque in Type-T mode for the transition B↔D. Here θ_0=-10°.

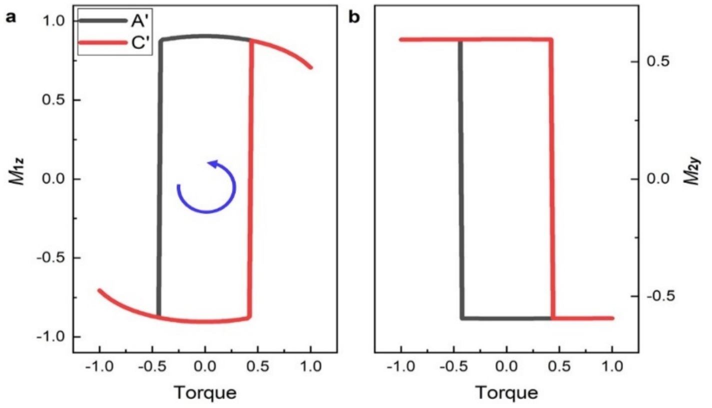

Supplementary Figure 10. The switching chirality when the titling angle is +10°. (**a**) and (**b**) indicate switching curves of M_{1z} and M_{2y} driven by spin-orbit torque in Type-T mode for the transition A'↔C'. Here θ_0=+10°. State A' and C' is slightly changed from State A and C due to the change in θ_0.

It will be helpful to switch some parameters such as *A* or EA tilting angle θ_0 off in the model to investigate their roles in Type-T switching with the other parameters unchanged. If θ_0=0°, switching behaviors are presented in Supplementary Fig. 11.

Supplementary Fig. 11a shows system has to pass through State D if it is initialized from State A and destinated to State C with negatively increasing torque. However, once the system goes into State D, we have to adopt Supplementary Fig. 11d to further analyze switching order since the initial state has been changed to State D. Supplementary Fig. 11d shows that the system will persistently remain in State D with further negatively increasing torque. This means the transition A↔C is actually forbidden dynamically while the transition A↔D is permitted. Similar arguments based on Supplementary Fig. 11b and c show the transition B↔D is also forbidden while the transition B↔C is permitted. Thus this results clearly show that large enough I_x in a system with θ_0=0° can switch the IMA layer only with the PMA layer retaining its M_z component during the whole process.

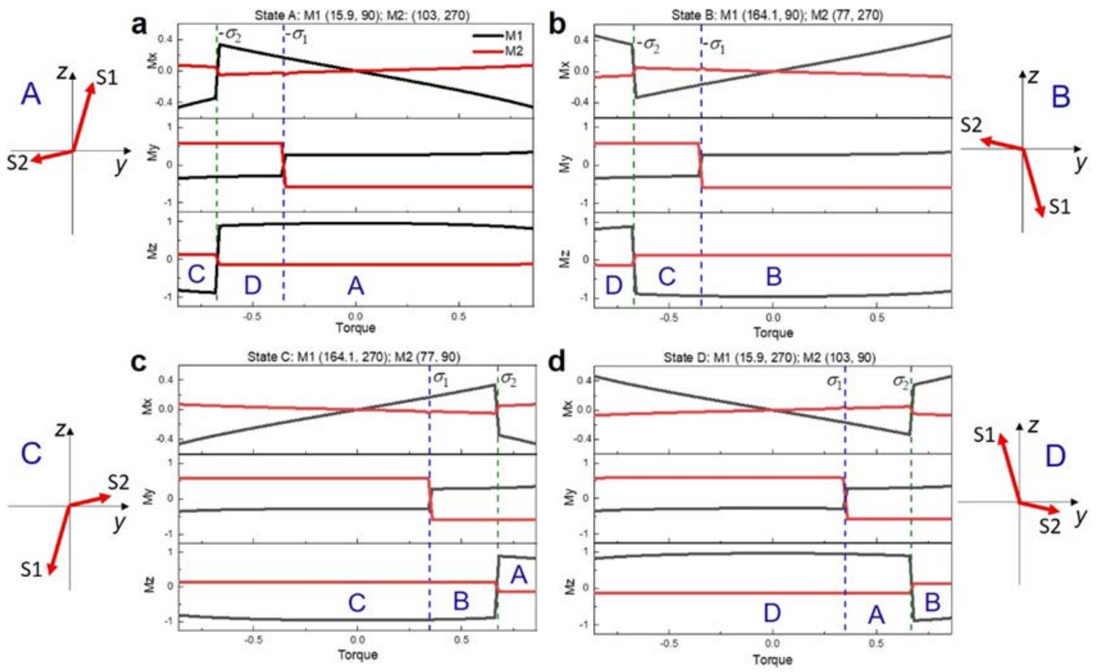

Supplementary Figure 11. Final states evolved from 4 different initial States A-D. (**a-d**) show the final state of A-D whose configurations are shown by the corresponding insets. Here, θ_0=0°. States A-D are thus recalculated.

If A=0, the IMA is still switchable with a larger critical switching current than in Type-T mode (Supplementary Fig. 12b). More importantly, the PMA layer can be only rotated in-plane instead of 180° deterministic switching as shown by Supplementary Fig. 12a.

Therefore interlayer coupling and easy axis tilting of PMA layer are both indispensable in this Type-T mode.

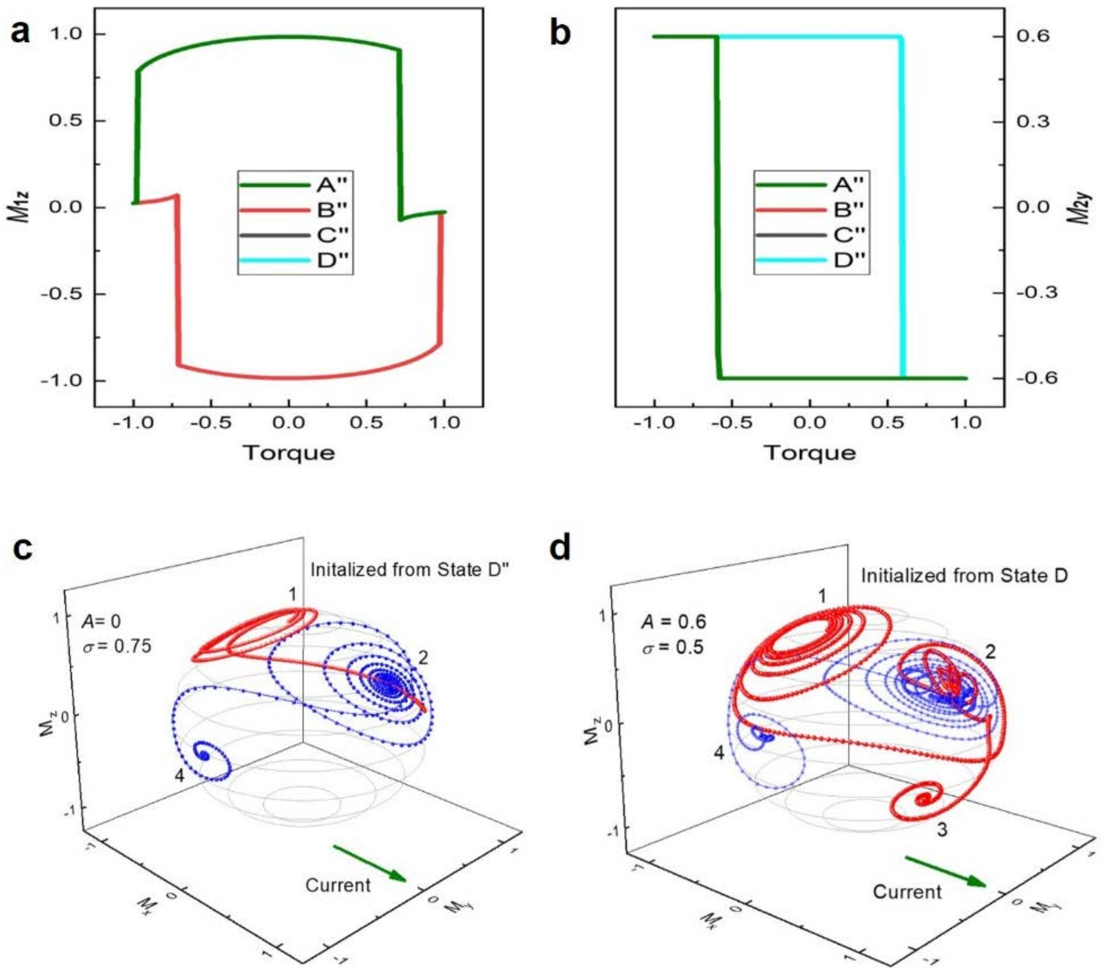

Supplementary Figure 12. Dynamic switching simulation results of Type-T mode. (**a**-**c**) Switching curves as A=0 and θ_0=-10° with the other parameters same with those in Supplementary Figure 2. While the PMA layer can be only rotated in-plane (**a**), only the IMA layer is switchable (**b**). Here the equilibrium States are slightly deviated from State A-D due to zero A. Normalized switching orbitals as (**c**) A=0 and σ=0.75 and (**d**) A=0.6 and σ=0.5. Spin dynamics are initialized from State D'' in (**c**) or State D in (**d**). If A=0, PMA layer can only reach an in-plane place around Point 2 (**c**). If A>0, the PMA switches its M_z component at the same time when the IMA switches its M_y component (**d**).

Spin dynamics with A=0 and A=0.6 (Supplementary Fig. 12c, d**)** indicates the switching

mechanism of Type-T mode. System initially locates at State D'' or D. In a system without coupling (A=0), a large I_x (or $+\sigma_y$) can drive magnetization of the PMA layer from Point 1 to an in-plane position around Point 2. In this case, $\mathbf{M}_1$ is nearly parallel with $+\sigma_y$. Instead, the IMA layer absorbs spin current with opposite direction ($-\sigma_y$), which is thus switched from Point 2 to Point 4. If the coupling is switched on (A=0.6), $\mathbf{M}_1$ is still first tilt to a location near Point 2. Owing to antiferromagnetic coupling, system becomes very unstable when $\mathbf{M}_1$ and $\mathbf{M}_2$ both parallelly locate near Point 2. Thus once $\mathbf{M}_2$ switches, system relaxes to its lowest energetic state at Point 3, State B with M_{1z} pointing down. It is the reason why M_{1z} and M_{2y} switch simultaneously in this mode as in Supplementary Fig. 8d2, d3.

Supplementary Note 4: Immunity of Type-T mode to external fields

We have also experimentally studied switching behaviors of Type-T mode under different $H_{x/y}$. The results in Figure 3-4 of main text show switching direction of Type-T mode cannot be changed by the external fields, which is remarkably different from the behavior of Type-z mode. Furthermore, H_y can offset M_{1z} vs. torque loops. These features can both be reproduced by the above time-dependent LLGS model as shown in Supplementary Fig. 13-14

Especially, M_{1z} switching is highly correlated with M_{2y} switching as shown above. They switch at the same critical torque. Now an external H_y is applied along the easy axis of the in-plane layer. On the basis of a uniaxial anisotropy, the in-plane layer obtains additional unidirectional anisotropy. This unidirectional anisotropy will make M_{2y} switching in one direction easier while in the opposite direction harder, thus offsetting the corresponding M_{2y} vs σ curves. Since M_{1z} switching depends on the M_{2y} switching, an offset would also occur in the corresponding M_{1z} vs σ curves as shown in Supplementary Fig. 14 which qualitatively reproduces the feature of Figure 4 of the main text.

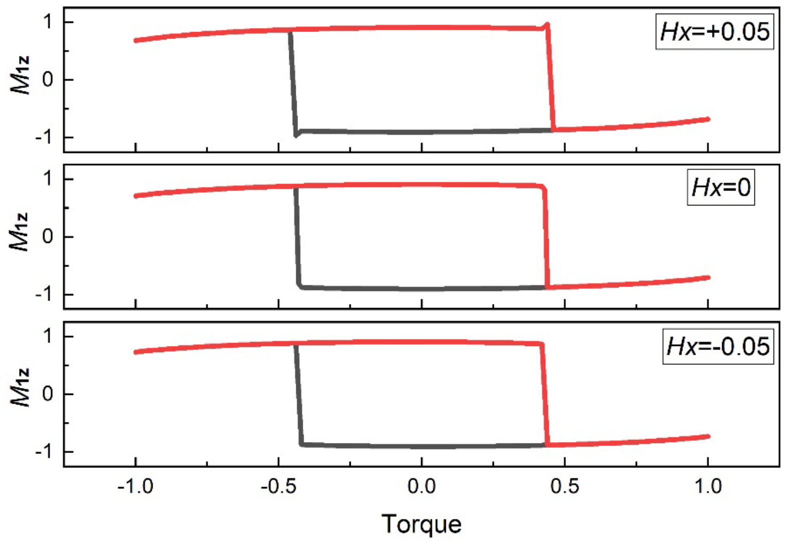

Supplementary Figure 13. Calculated M_{1z} vs. torque hysteresis loops under different H_x. Switching direction cannot be changed by H_x.

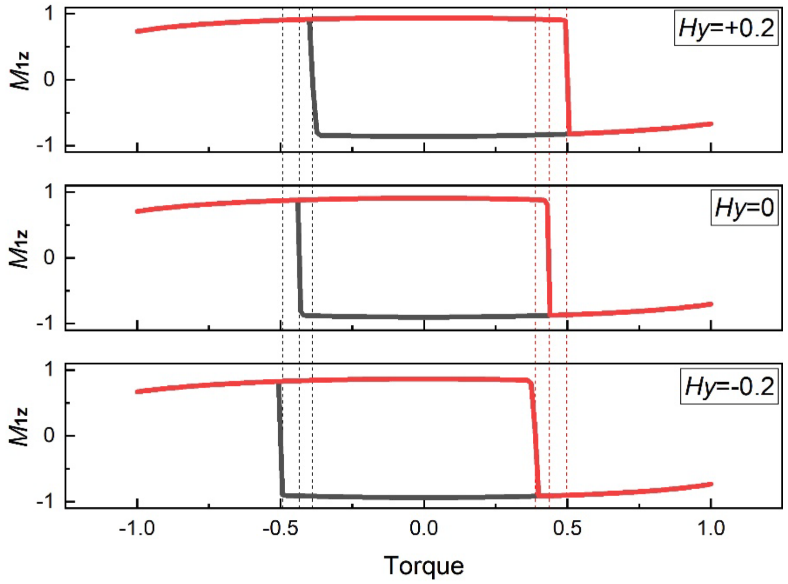

Supplementary Figure 14. Calculated M_{1z} vs. torque hysteresis loops under different H_y. H_y can offset the loops by affecting switching of IMA layer which is a preliminary condition to switch PMA layer since the two layers switch simultaneously at the same critical torque.

We give a comparison between Type-T and Type-z modes in Supplementary Table 1 as a summary.

Supplementary Table 1. Thorough comparison between Type-T mode and Type-z mode based on the above calculations. It is notable that the parameters for calculation are the same with that in Supplementary Fig. 2.

Field	Type-T mode	Type-z mode
Switching layers	IMA and PMA layers switch at the same torque	Only PMA
Material requirement	$A \neq 0$, EA tilting of PMA layer toward EA of IMA layer	$A \neq 0$
Results of EA tilt of PMA layer	Indispensable for Type-T mode	Offset M_{1z} vs. torque loops
Switching direction	Determined by EA tilting angle	Determined by direction of M_{2y}
H_x Sensitivity	Insensitive	Change switching directions
H_y Sensitivity	Offset M_{1y} vs. torque loops	Making switching harder
Transition Route	B↔D as $\theta_0<0$ and A↔C as $\theta_0>0$	A↔B as $M_{2x}<0$; C↔D as $M_{2x}>0$
Advantages	Robust immunity to external fields with a fixed switching direction	Controllability of switching directions by fields and M_{2x}
Significance	(1) Demonstrate a new scheme to switch PMA films with high robustness<br>(2) Develop a MRAM compatible structure to support two switching modes<br>(3) Benefit for MRAM, multistate memory and spin logic applications	

Supplementary References:

1. Wang X., *et al*. Field-free programmable spin logics via chirality-reversible spin–orbit torque switching. *Adv. Mater.* **30**, 1801318 (2018).

2. Baek, S. C., *et al*. Spin currents and spin-orbit torques in ferromagnetic trilayers. *Nat. Mater.* **17**, 509 (2018).
